# Recent Advances in the Characterization of Subsurface Damage in Optical Materials

**DOI:** 10.3390/ma18163883

**Published:** 2025-08-19

**Authors:** Liwei Ou, Hongtu He, Fang Wang, Laixi Sun, Jiaxin Yu

**Affiliations:** 1 Key Laboratory of Testing Technology for Manufacturing Process in Ministry of Education, Southwest University of Science and Technology, Mianyang 621010, China; lwou@swust.edu.cn (L.O.); yujiaxin@swust.edu.cn (J.Y.); 2 Research Center of Laser Fusion, China Academy of Engineering Physics, Mianyang 621900, China; shilan_wang@126.com

**Keywords:** optical materials, subsurface damage, characterization methods, destructive and non-destructive

## Abstract

Subsurface damage (SSD) in optical materials is easily induced during ultra-precision processes and plays a critical role in the surface performance and lifetime of optical materials. Without proper characterization of SSD in optical materials, it is difficult to fully understand its properties across various manufacturing processes and thus find effective ways to eliminate it. Despite the rapid development of SSD characterization, many unique features, principles, and applications of SSD characterization in optical materials are not known to this community. This review systematically reviewed the recent literature on characterization methods of SSD in optical materials in both destructive and non-destructive ways. The major drawbacks and limitations of all the characterization methods are presented in this paper, and future trends in the characterization of SSD in optical materials are also proposed.

## 1. Introduction

Owing to their superior optical, mechanical, and chemical properties, optical glasses and crystals have been widely used as key components in various industries, the military, and scientific research applications. For instance, optical glasses are the key substrate materials for optical windows [1] and display screens [2], as well as optical lenses [3] and intense laser components [4]. Optical crystals, such as CsB_3_O_5_ and K_3_YB_6_O_12_, have been widely used in laser imaging [5], environmental monitoring [6], laser medicine [7], chemical remote sensing [8], and infrared countermeasures [9]. All applications of typical optical components rely on the surface and subsurface quality after ultra-precision processing involving cutting and lapping as well as grinding and polishing; however, subsurface damage (SSD) of optical materials can easily occur due to the physical contact with abrasive and polishing particles during these ultra-precision processes, even though the surface roughness can be substantially reduced by precise material removal [10]. It is well known that the presence of an SSD layer will significantly reduce the strength or lifetime of an optical material [11,12] and the quality of functional coating during subsequent surface treatment [13]. Thus, it is necessary to characterize the SSD of optical materials after super-precise and subsequent surface treatments, because this not only provides direct information on the size, type, location, and spatial distribution of SSD but also provides guidelines for subsequent machining and surface treatment to remove the SSD layer to achieve better machining quality and efficiency.

**Figure 1:**
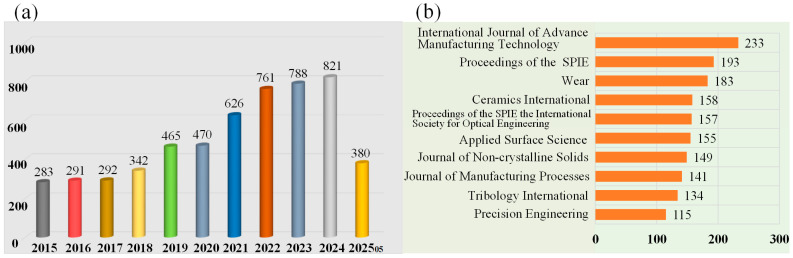
Literature survey results for the (**a**) research articles and (**b**) journal publications available on the SSD characterization of optical materials in the last ten years. Source: Web of Science.

Figure 1a presents a literature survey quantifying research articles on the SSD of optical materials over the past decade. Annual research output consistently exceeds 200 articles, demonstrating a clear upward trajectory. Based on our comprehensive survey, we assert that the majority of relevant work during this period has been captured. These findings highlight a significant emerging research focus on SSD in optical materials while simultaneously revealing a persistent gap in fundamental understanding within the existing body of work. We also note that the top publications are *International Journal of Advanced Manufacturing Technology, Proceedings of the SPIE, Wear, Ceramics International, Applied Surface Science, Journal of Non-Crystalline Solids, Journal of Manufacturing Processes, Tribology International, Precision Engineering*, etc. (Figure 1b). Our review aims to concisely trace the evolution of SSD in optical materials, from its historical origins to current advancements. We further discuss the diverse experimental methodologies employed to investigate SSD in optical materials, elucidating the specific value of each technique. Consequently, our primary goal is to synthesize the current comprehension of SSD in optical materials and delineate pivotal unresolved questions, thereby offering a foundation for future investigations of SSD in optical materials.

## 2. Mechanism of Subsurface Damage Formation

Generally, optical materials are hard and brittle materials; thus, there are many types of SSD in optical materials, such as cracks, residual stress, porosities, phase transformation, and amorphization [14,15]. Among all subsurface damage, cracks are the most detrimental factor and can directly determine the performance and lifetime of an optical component. During precision processes such as grinding and polishing, the fundamental mechanism of surface material removal is based on direct contact of the hard indenter or polishing particles under an externally applied load. As early as the 1970s, Lawn et al. [16] proposed the classical fracture mechanics model during static indentation, where a plastic deformation zone can be found underneath the static indenter, and medium cracks form parallel to the material’s surface [17] while radial cracks are formed perpendicular to the material’s surface [18]. Medium crack propagation and fracture are the most effective methods of material removal during the grinding and polishing process [19,20], while radial cracks and Hertzian cracks are the main elements of SSD in brittle materials [21]. Based on the fracture mechanics model, Wang et al. [22] developed a critical function for crack propagation during single grit scratching of fused silica and predicted that the material removal mode will evolve from a ductile mode to a semi-brittle mode, a full-brittle mode, and a semi-brittle mode in sequence with increasing single grit scratching depth. The brittle mode of a material will occur when the normal load applied on the indenter or particle is higher than the critical load *P_m_* [23]:

(1) $$P_{m}=\left(\frac{54.57\alpha }{\beta^{2}\theta^{4}}\right){\left(\frac{K_{c}}{H}\right)}^{3}K_{c}$$

where *α* is a dimensional factor determined by the indenter geometry, *β* and *θ* are the dimensional factors determined by the machining condition, and *K*_c_ and *H* are the fracture toughness and hardness of the material, respectively.

So far, several models have been proposed to calculate the depth of medium cracks. For instance, Jing et al. [24] proposed an improved blister field model for the scratch process, where the blister field strength is explicitly determined using the material properties, loading conditions, and geometry of the scratch tool. The depth of medium cracks can be expressed as:

(2) $$c_{1}={\left[\frac{3\left(1\;-\;2v\right)}{5\;-\;4v}+\frac{2\sqrt{3}}{\pi \left(5\;-\;4v\right)}\frac{E}{\sigma_{y}}\cot\varepsilon \right]}^{1/2}\tan\alpha h_{i}$$

where *E* is the elastic modulus, *v* is Poisson’s ratio, *σ_y_* is the yield stress, and *ε* and *h_i_* are the semi-vertical angle and penetration depth of the grits, respectively.

Another study by Gu et al. [25] found a correlation between the depth of subsurface cracks and scratch depth during scratch tests of BK7 glass, and the depth of medium cracks can be expressed as:

(3) $$c_{1}=0.206\frac{{\left(E\cdot H\right)}^{1/3}}{{\left(Kc\cdot B\right)}^{2/3}}{(\cot\alpha )}^{4/9}{(\tan\alpha )}^{4/3}h_{i}^{4/3}$$

where *B* is a material parameter determined by the elastic recovery during the scratch process.

In another study by Suratwala et al. [26], based on substantial scratch tests and SSD depth measurements from a wide range of optical materials, semiempirical relationships were formulated to estimate the SSD depth as a function of workpiece material and important grinding parameters:

(4) $$c_{1}={\left(\frac{\chi_{h}P}{Kc}\right)}^{2/3}$$

where *P* is the normal load during the scratch process, and χ*_h_* is a dimensional factor determined by machining conditions.

**Figure 2:**
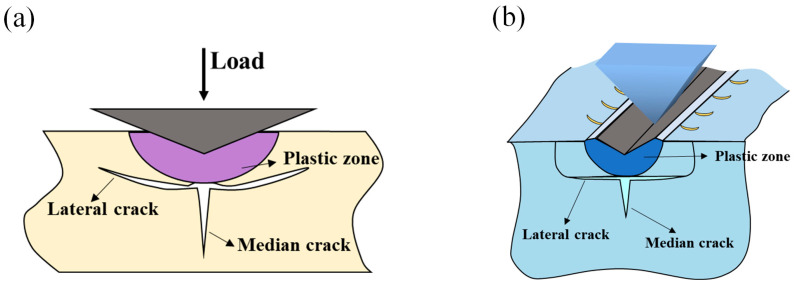
Crack formation mechanisms upon (**a**) indentation and (**b**) scratch tests.

As the grits or particles slide or roll on an optical material’s surface, not only the normal load but also the tangential force that is parallel to the movement direction can affect the SSD, where some tailing cracks (which are observed as arc defects at the macroscale) can be found [27] (Figure 2b). Furthermore, Suratwala et al. [28] found the depth of tailing cracks in fused silica after grinding can be expressed as:

(5) $$c_{1}={\left(\frac{\chi_{h}{\left(1+\mu^{2}\right)}^{2}P}{Kc}\right)}^{2/3}$$

where *μ* is the friction coefficient of the material.

## 3. Characterization Methods of SSD

**Figure 3:**
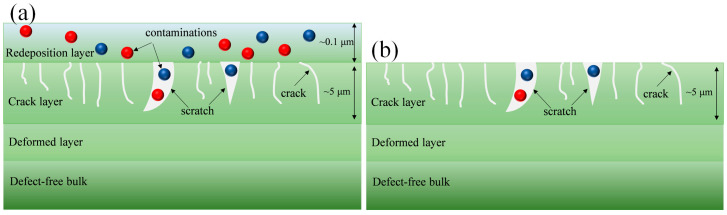
Typical SSD in optical materials after machining in (**a**) ductile regimes and (**b**) brittle regimes.

During the mechanical machining process, various types of surface and subsurface deformation of optical materials can occur depending on the contact conditions, including applied load and speed, as well as the physical and shape of grits or particles. At the macroscale, scratching, fracturing, and peeling of optical materials can occur, while at the microscale, microcracking and crystalline structure evolution can be identified as SSD. At the nanoscale, various types of SSD can be detected, such as porosity, chemical structure defects involving oxygen-deficient centers (ODCs) and non-bridging oxygen hole centers (NBOHCs) in glass, contaminants in the Beilby layer, and/or new contaminants from the subsequent surface treatment procedure. Typical SSD in optical materials after polishing is shown in Figure 3a. The SSD layer can differ depending on the machining procedure: an elastic–plastic deformation layer can be found in the SSD layer when it is machined in a ductile regime, while a subsurface cracking layer and deformed layer exist when it is machined in a brittle regime (Figure 3b).

**Figure 4:**
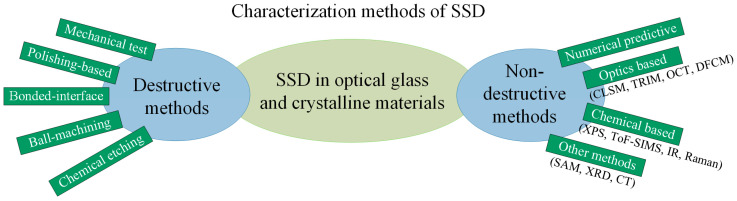
Overview of SSD characterization methods in optical materials.

For SSD in optical materials, various characterization methods have been developed and widely used in academia and industry, and those methods can be classified into two categories: destructive and non-destructive methods. An overview of the characterization of SSD in optical materials is given in Figure 4. In the following sections, these methods are compared and discussed.

### 3.1. Destructive Methods

#### 3.1.1. Mechanical Test Methods

Since defects or damage in the chemical structure will affect the mechanical properties of optical materials, mechanical property measurements can reveal SSD in optical materials to some extent. For instance, using the micro-indentation technique, Yang et al. [29,30] determined that the near-surface mechanical properties of single-crystalline silicon and glass were reduced due to the presence of SSD introduced during the lapping process, where the reduced modulus decreased with increasing SSD, while the residual indentation depth increased with SSD. Similarly, Ma et al. [31] utilized nanoindentation technology and found that the gradient change of the elastic modulus of fused silica is consistent with the evolution of SSD at various depths, but such consistency is not found when for the nanohardness. Later, Wang et al. [32] found that the nanomechanical parameters (hardness and elastic modulus) of the subsurface layer in borosilicate (BK7) glass followed an exponentially descending trend with the shortening of distance from the lapped surface, which was consistent with the increase in crack density. In another study by Guo et al. [33], it was found that the nanoscratch resistance of quartz glass varied depending on the SSD depth. In addition, numerous experiments and molecular dynamics simulations have shown that the nanohardness and modulus of oxide glass increased with subsurface densification [34,35,36], while they decreased with the presence of subsurface cracking [37,38]. It can be seen that mechanical property measurements strongly depend on the thickness and distribution of SSD, but multiple types of SSD can exist at the same time, thus future analyses of SSD via mechanical property measurements should be focused on the relationship between various types of SSD and their mechanical properties.

#### 3.1.2. Polishing Methods

**Figure 5:**
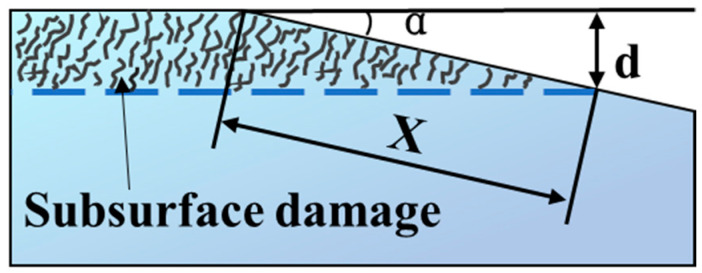
Schematic drawing showing traditional taper polishing.

As one of the widely used methods, the traditional taper polishing method is based on the direct observation of exposed SSD on the wedge after cutting a machined sample along angle α, along with the subsequent polishing and etching processes [39,40]. Based on the distribution of SSD on the wedge, the SSD depth d can be expressed as (Figure 5):

(6) d=x·sinα

where *x* is the length of SSD on the wedge, and α is the angle of the wedge to the pristine surface. It should be noted that the measurement error of traditional polishing methods can be relatively high because new damage can be introduced to the sample, and the SSD region could be enlarged to some extent, although they can provide direct, accurate, low-cost, and easy-handling methods [41].

In contrast to the traditional polishing method, magnetorheological finishing (MRF) is a precise process where no extra damage is introduced into the optical components [21,42,43]. The SSD distribution can be determined by measuring the width of the spot and/or wedge, with the depth usually being tens of micrometers, while the width is in the order of millimeters. Since the depth is magnified hundreds of times by this method, it can improve accurate measurements. It should be noted that polishing-based methods can provide local SSD information only, and global information and the spatial distribution of SSD cannot be determined. In addition, the SSD may be overestimated because subsequent polishing or etching processes can enlarge pristine crack lengths. Thus, future studies of those methods should be focused on providing more global information with higher measurement accuracy.

#### 3.1.3. Bonded-Interface Methods

**Figure 6:**
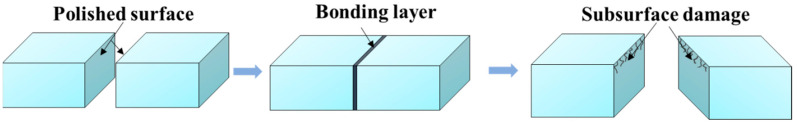
Schematics of the bonded-interface technique.

First proposed by Mulhearn et al. [44] to investigate the indentation-induced material deformation of metals, bonded-interface techniques (BITs) can also be used to reveal SSD of optical materials [45]. Generally, BITs contain three steps: firstly, polish two surfaces and then bond the two polished surfaces together; secondly, machine the work surface after bonding the polished surfaces; then, remove the bonding glue and observe the SSD on the two polished interfaces directly using optical microscopy (Figure 6). BITs possess restricted applicability in characterizing SSD within ceramics, optical glasses, and comparable hard, brittle substances. This restriction originates from the erroneous representation found in the resulting stress–strain field data [20,46,47].

A fundamental limitation of BITs arises from the bonded layer within the specimen. This layer disturbs the local stress–strain fields adjacent to the interface during indentation loading. Through combined finite element simulations and indentation experiments, Helbawi et al. [48] demonstrated a shift in the maximum stress location away from bonded interfaces. Consequently, the peak SSD depth occurred at a greater distance from the interface and exceeded the measured damage depth [48]. Nevertheless, BIT observations revealed a damage zone with higher density than typically observed in actual machining processes. These findings suggest that BITs may not offer precise data on SSD generated during practical machining and may be unsuitable for the quantitative assessment of SSD in hard, brittle materials.

Based on cross-section samples prepared using BITs or similar methods, the SSD of optical materials during mechanical processing can be directly characterized. For instance, using scanning electronic microscopy (SEM) and cross-section sampling of glass materials, Bandyopadhyay et al. [49,50,51], He et al. [52,53], and Feng et al. [54] found the SSD of optical glasses was strongly dependent on the loading conditions (applied load, scratch speed, tool apex angles) and environments during the scratch process. On the other hand, using transmission electron microscopy (TEM) and cross-section samples of optical materials, SSD defects can be precisely characterized as microcracks [55], dislocations [56], amorphous, or multi-crystalline [57], as well as other micro- and nanoscale defects [58,59]. Since SEM and TEM can observe microscale damage at a higher resolution than optical microscopy, they can provide a more comprehensive estimation of SSD in optical materials. It should be noted that TEM requires the sample to be thin enough due to the high scattering and poor penetration into the sample surface by electrons, and samples need to be thinned to 20–50 μm, and then further to less than 1 μm by ion milling [60,61]. Therefore, the cross-section sample preparation procedure in a TEM observation is complicated and time-consuming. In addition, SEM and TEM observations of SSD in optical materials cannot provide global SSD information.

#### 3.1.4. Ball-Machining Methods

**Figure 7:**
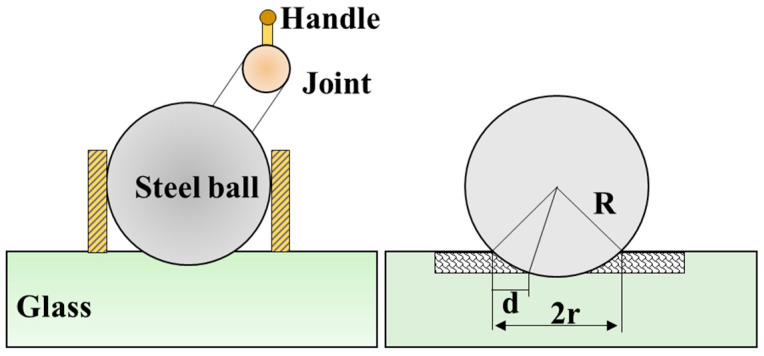
Schematics of the ball-machining technique.

Traditional ball-machining methods are based on the indentation of a steel ball on an optical material’s surface, where the SSD can be characterized from a side view of the indentation when the indentation depth exceeds the SSD depth (Figure 7) [62]. Such methods are easy to operate, but some new SSD can be introduced during the indentation process, and subsurface crack propagation can be observed due to the applied force in the surface normal direction. Thus, the measurement error of ball-machining methods is relatively high.

Magnetorheological finishing (MRF) methods can remove materials based on shear effects without introducing new damage during polishing, so a MRF-based ball-machining method is widely used in the local characterization of SSD in fused quartz. The basic principle is to use MRF technology to create speckled pits on the fused quartz surface, and the SSD can be exposed with the aid of HF etching. Depending on the transverse size of the subsurface crack and the contour of the magnetorheological pits after HF etching, the pits can act as subsurface crack amplifiers that can translate the measurement of micron-scale crack depth into millimeter-scale measurement of horizontal crack growth [63]. Unlike traditional ball-machining methods, MRF methods do not introduce new mechanical damage. The action area is smaller, the operation process is relatively simple, and the working cycle is short, which is more suitable for rapid characterization of the local subsurface defect morphology of brittle materials compared with magnetorheological wedge polishing methods.

#### 3.1.5. Chemical Etching

Chemical etching is a widely used method that uses a suitable etchant to remove the damaged layer of a machined sample. The surface morphology and roughness after etching, as well as the etching rate, can be used to evaluate the depth of SSD. Currently, there are several methods for estimating the SSD of optical materials using chemical etching.

Selective etching is reported to remove SSD to improve surface quality [64,65]. Recently, Wu et al. [66] utilized HF/HNO_3_ mixtures to identify ultrathin friction-induced amorphous damage on a Si surface using diamond tip scratching. The effects of normal load during the friction process, etching time, and volume ratio of HF/HNO_3_ mixtures on selective etching were investigated to optimize parameters for amorphous Si detection. The selective etching method can provide high resolution, high efficiency, and low destruction; however, it has limited application in characterizing subsurface cracking and deformation layers.

Another method is based on the evolution of surface roughness during various etching processes. Generally, SSD areas have different chemical reactivity compared to damage-free areas, and thus they have different etching rates and mechanisms compared to damage-free optical materials. For instance, during the HF etching of fused quartz, Wong et al. [67] found that the peak-to-valley (PV) value and root-mean-square deviation (*R*_q_) of fused quartz varied with etching time. They found that subsurface cracks will be exposed at the initial etching stage, which allows the probe tip to reach the inside of the crack, providing a high measurement value of surface roughness. With an increase in etching time, more subsurface cracks will be exposed and interact with each other, causing material removal. After the etching process, the total number of subsurface cracks will decrease, causing a decrease in surface roughness. The evolution of surface roughness with etching time can facilitate the calculation of SSD depth.

Other methods are based on etching rate, and constant- and differential-rate characterization methods are widely used to estimate SSD. The constant-rate etching method estimates the SSD depth by comparing the etching rates of the damaged layer and the damage-free layer, and it has been widely used to detect SSD in optical glasses, ceramics, and semiconductor materials [67,68,69]. However, the etching process can be influenced by many factors, such as etching time, etchant concentration, and temperature, which will cause a non-linear etching rate with time. Thus, it is difficult to precisely control all the influencing factors, and the measurement error for SSD depth could be relatively poor. To reduce the measurement error, differential-rate etching methods can be used by comparing the differential etching acceleration inflection points of the damaged layer and the matrix [70,71]. This method is easy to handle and low-cost, and it can provide in-depth information on subsurface cracking and deformed layers; however, the measurement efficiency is relatively low, and the measurement accuracy is in the micrometer range.

### 3.2. Non-Destructive Methods

Destructive characterization techniques necessitate partial or complete sample destruction, depending on the specific method employed. This inherent requirement renders them time-consuming, reduces characterization efficiency, and elevates overall costs. Conversely, non-destructive approaches preserve sample integrity. These methods leverage principles involving light beams, electromagnetic waves, acoustics, etc., offering significantly higher efficiency and lower operational costs.

#### 3.2.1. Numerical Predictive Methods

Numerical predictive approaches primarily focus on estimating the SSD depth, as opposed to direct measurement. These methods generally fall into two categories: those utilizing machining process parameters and those leveraging the surface roughness (*SR*) of the finished sample. Historically, empirical predictions of SSD depth have relied on the size of abrasive grains [72,73]. For instance, Lambropoulos et al. [74] established a predictive correlation between SSD depth (*d*_SSD_) and abrasive grain size (*d*):

(7) $$0.3d^{0.68}<d_{SSD}<2d^{0.85}$$

Based on both the grinding parameters and material properties, Zhang et al. [75] built a predictive model to predict the SSD depth (*d*_SSD_) of ceramics:

(8) $$d_{SSD}={\left(200a_{g}\right)}^{\frac{1}{log(\lambda \frac{H}{K_{c}})}}$$

where *λ* is a constant (10^−2^ *m*^1/2^, *m* is the working condition coefficient, 1/2 < *m* < 2/3), *H* and *K_c_* are the hardness and fracture toughness of a workpiece, respectively, and *a_g_* is the maximum cutting depth of the abrasive grains.

On the other hand, the *SR* after machining can also be used to predict the SSD depth, which is derived from fracture mechanics. As one example, in studying lapping of glasses and ceramics with SiC abrasives, the ratio of *SR* and SSD depth was found to be 3.9 ± 0.2 [21]. However, this ratio can differ depending on workpiece materials, machining processes, and SSD measurement methods [21,76,77]. Based on indentation fracture mechanics, Lambropoulos et al. [78] and Randi et al. [43] proposed an explicit equation to predict SSD depth (*d*_SSD_):

(9) $$d_{SSD}={2.33\alpha_{k}^{\frac{2}{3}}\left(\frac{E}{H}\right)}^{\frac{2-5m}{3}}\frac{{\left(cot\varphi \right)}^{1/9}}{{\left(sin\varphi \right)}^{1/2}}{\left(\frac{P}{K_{c}^{4}/H^{3}}\right)}^{1/6}SR$$

where *d_SSD_* and *SR* are the SSD depth and surface roughness, respectively. *E*, *H*, and *K_c_* are the elastic modulus, hardness, and fracture toughness of the workpiece, respectively. *φ* is the semi-angle of the sharp cone of an abrasive grain. *P* is the applied force acting on the grain. Depending on the force *P*, *α_K_* and *m* are numerical factors in the ranges of 0.03–0.04 and 0.33–0.50, respectively. To eliminate the effect of force *P*, Li et al. [79] proposed a numerical relationship between *d_SSD_* and surface roughness (*SR*):

(10) $$d_{SSD}=3.08{\left(k\alpha_{k}\right)}^{\frac{2}{3}}\frac{1}{{\left(sin\varphi \right)}^{2/3}}\frac{H^{2m}}{E^{\left(2m-\frac{2}{3}\right)}K_{c}^{2/3}}{SR}^{4/3}$$

It should be noted that all these methods are based on indentation fracture mechanics, where the effect of the cutting speed of the tool is ignored. To study the effect of cutting speed, Li et al. [80] proposed relationships between the *d_SSD_* and the grinding wheel speed and workpiece feed rate of BK7 glass:

(11) $$d_{SSD}=\lambda \times {\left(\frac{v_{w}\;\times \;L}{v_{s}}\right)}^{4/3}{\times [arccos(2\sqrt{a/d})]}^{4/3}-\frac{v_{w}\;\times \;L\;\times \;arccos(2\sqrt{a/d})}{v_{s}\;\times \;sin[\arccos\left(2\sqrt{a/d}\right)]}$$

where *λ* represents a parameter influenced by the workpiece material properties and abrasive grain geometry, and *v*_w_ and vs denote the workpiece and grinding wheel velocities, respectively. *L* signifies the spacing between successive cutting edges, and *a* and *d* correspond to the depth of the cut and the grinding wheel diameter. Critically, this model incorporates an assumption of constant grit protrusion height—a condition diverging from real-world grinding scenarios. It is significant to note that while existing numerical predictive models offer a relatively straightforward approach for estimating SSD depth, they consistently fall short in accurately predicting the maximum SSD depth. The deepest damage zone poses the greatest threat to optical component performance and longevity. Additionally, subsurface cracks possess the potential to propagate significantly beyond initial depths, meaning predictions reliant solely on surface roughness data may incur substantial errors. [81]. Furthermore, these predictive methods predominantly stem from Lawn et al.’s quasi-static indentation fracture framework [82]. However, actual machining conditions frequently deviate from this model’s premises, particularly with the prevalent use of high cutting speeds. Consequently, incorporating the effects of temperature and strain rate on material behavior is essential for advancing future SSD prediction models [83].

In a recent study, Yin et al. [84] performed a series of grinding experiments on K9 glass, systematically varying key processing parameters such as abrasive grain size, grinding depth, wheel speed, and feed rate. Leveraging a genetic algorithm (GA) and a deep neural network, they established a predictive model linking the processing parameters *Ra*, *Sa*, and SSD depth. This model demonstrated high accuracy and reliability, achieving a mean absolute percentage error below 10% and a correlation coefficient exceeding 0.94. These findings highlight the potential of machine learning as a cost-effective and efficient approach for predicting both SSD depth and surface topography in brittle materials based on machining inputs. Furthermore, the integration of AI-assisted prediction models and deep learning for image recognition will further enhance the accuracy, efficiency, and scalability of SSD detection. Significant potential lies particularly in three key areas: enhancing accuracy and resolution via deep learning, addressing data scarcity and complexity, and enabling real-time monitoring and automation.

#### 3.2.2. Optics-Based Methods

Optics-based SSD detection technology quantitatively estimates SSD by detecting the difference between the characteristic optical parameters of the damaged layer and the substrate and has been widely used for defect detection in metals [85], ceramics [86], and optical materials [20,87,88]. The depth of SSD depends on the machining process, and the SSD depths of optical materials from grinding and polishing are within the range of several micrometers and tens of micrometers, and some optics-based SSD detection techniques can satisfy their measurement, such as confocal laser scanning microscopy (CLSM), total internal reflection microscopy (TRIM), optical coherent tomography (OCT), etc.

**Figure 8:**
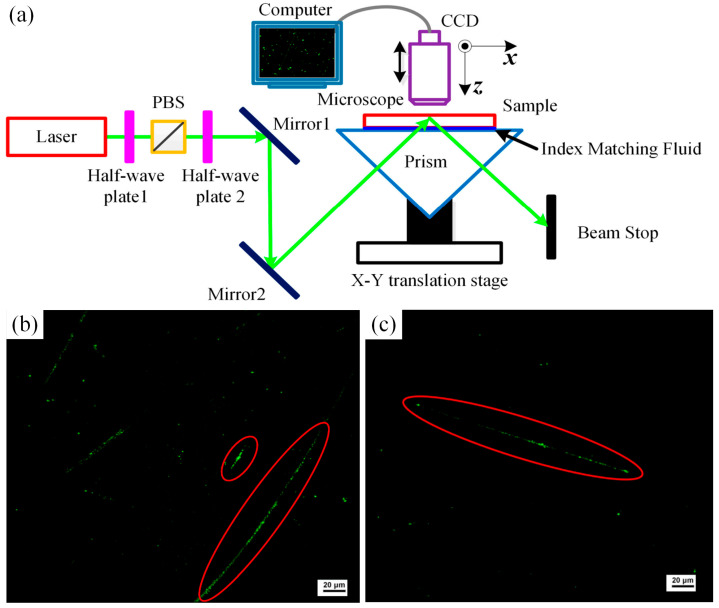
(**a**) Schematic of the improved TRIM setup and (**b**,**c**) Typical image of SSD in fused silica obtained using TRIM [94].

First proposed by Temple et al. [89], total internal reflection microscopy (TIRM) has been used to detect surface/subsurface damage of optical materials [90,91]. The working principle is shown in Figure 8a. The test sample is placed on top of a prism with a matching fluid in between, and a linearly polarized light passes through the prism and the matched fluid at an angle of incidence equal to or slightly greater than the critical total reflection angle, illuminating the sample. If there are no defects on the surface and subsurface of the sample, total reflection occurs at the interface between the sample and the air, no light is scattered into the microscope, and the lens captures a type of full dark-field image; in contrast, if the test sample contains surface or subsurface defects, part of the incident light will be scattered, and the outgoing defect scattered light will be received by the microscope to obtain a scattering image (Figure 8b,c). Liao et al. [92] utilized TRIM to detect the SSD of fused silica and found that TRIM images can be applied for SSD detection. Xu et al. [93] and Ni et al. [94] established a relationship between the definition of TIRM images and the depth of the SSD of fused silica using an improved TRIM method, where the position and extent of SSD along the depth direction could be acquired simultaneously. It should be noted that surface roughness can significantly affect TRIM results; thus, the TRIM technique requires samples with low surface roughness and transparency to the incident light [20], and it can only provide qualitative information about SSD.

**Figure 9:**
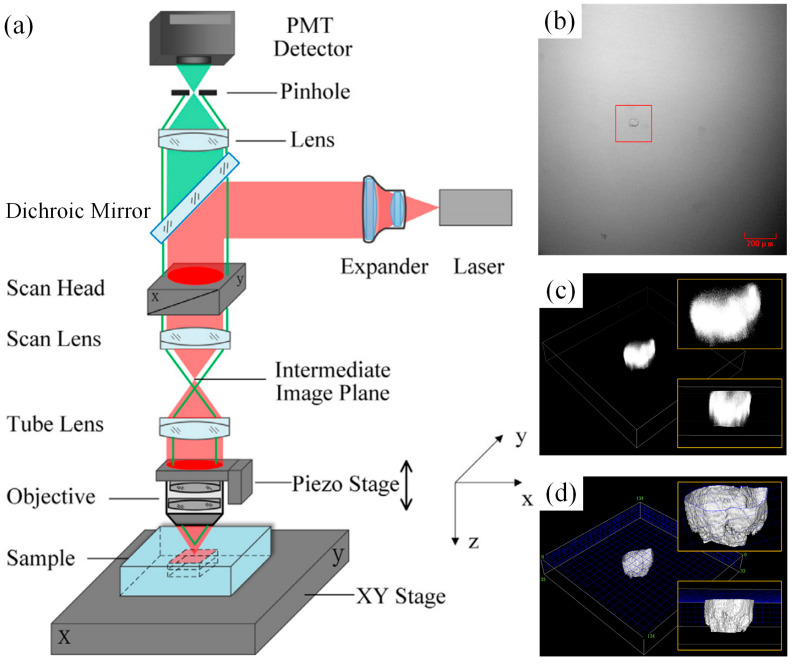
(**a**) Principles of CLSM for SSD detection, (**b**) images of an open crack in a bright field micrograph, (**c**) 3D reconstruction results from an NIS-Elements Viewer, and (**d**) 3D reconstruction results obtained using the modified algorithm [99].

Confocal microscopy offers superior detection resolution compared to traditional microscopy, leading to its widespread adoption for subsurface damage (SSD) characterization in ceramics [95], optical components [96], and semiconductors [97]. Figure 9a illustrates the operational principle of confocal laser scanning microscopy (CLSM). Within this system, a collimated and expanded laser beam enters the microscope and is directed by a dichroic mirror onto a galvanometer scanning head. Subsequently, the scanning lens, tube lens, and objective focus the laser onto the sample surface. Fluorescence emitted specifically from this illuminated focal point is collected by the objective, passes through the dichroic mirror and a confocal pinhole, and ultimately reaches the detector (e.g., photomultiplier tube). Crucially, only light originating precisely at the focal plane traverses the pinhole; signals from out-of-focus planes are physically blocked, enabling precise point detection. Winn et al. [95] utilized CLSM for SSD assessment in alumina, yet reported a maximum measurable depth limited to 20 µm. Conversely, Fine et al. [98] applied CLSM to polished and finely ground optical glass, achieving a resolution as fine as 150 nm. Bertussi et al. [96] found SSD in fused silica with resolutions of 1 μm in depth and 0.26 μm in the scanning plane using CLSM. More recently, Sun et al. [99] utilized 3D scanning of a small area using a galvo and piezo stage as well as CLSM and proposed a modified 3D reconstruction algorithm for subsurface defects with a low memory footprint and running time (Figure 9b–d). Note that the CLSM technique requires samples to have low surface roughness, and its resolution depends on the numerical aperture and wavelength [98]; thus, it is generally used for smooth and transparent materials.

Fluorescence confocal microscopy is a laser scanning confocal microscopy technique that introduces a fluorescence-enhancing medium into SSD. For instance, Neauport et al. [42] added a small amount of barium into slurry during grinding, tracked the barium at different etching depths, and inferred the depth and position of subsurface cracks. Similarly, Williams et al. [100,101] used “quantum dots”, nanometer-size fluorescent particles, to detect subsurface cracks in glass during lapping by detecting the intensity of the fluorescence emitted from the quantum dots trapped in the subsurface cracks. However, fluorescent particles may not penetrate into all SSD layers; thus, the SSD depth information obtained using this method could be less than the actual SSD depths [102].

Dark-field confocal microscopy (DFCM) is a promising technique for obtaining 3D information about defects compared to traditional inspection methods [103,104,105,106,107]. The basic principle and the layout of the DFCM method were explained by Liu et al. [108]. An annular beam focuses on the test position of the sample, exciting scattering of the light. The reflected or transmitted light follows the original path, and it is revealed as an annular beam, whereas the scattered light propagates in a random direction. The specular reflected beam from the surface propagates along the illumination path, which is removed by the complementary diaphragm. Meanwhile, the scattered beam from the SSD is collected by a detector with a pinhole mask to achieve dark-field confocal detection. In 3D dark-field confocal microscopy, a pair of axicons is employed to achieve size-adjustable and high-efficiency beam shaping. Liu et al. [104] used this setup to measure the SSD in neodymium–glass and eventually obtained 3D information about the SSD distribution within 60 nm depth in the subsurface. Huang et al. proposed a modified DFCM system by applying the optical fiber as a core element to generate an annular beam, which effectively compresses the system size and allows for high contrast imaging. To verify the reliability and accuracy, the glass surface morphology was accurately scanned with a high-resolution AFM, and it was found that most of the defects scanned by AFM were consistent with the results obtained using DFCM methods [109]. An edge occlusion imaging model based on a variable point spread function can lead to higher accuracy of the imaging, reducing the deviation by 92.71% and the standard deviation by 57.58% [110]. The advantage of DFCM is the ability to obtain depth information and the 3D quantitative distribution of SSD while keeping the axial tomographic capability of confocal microscopy. This method can detect nano/micro-surface scratches and abrasions, making it additionally suitable for rough surface measurements.

Optical coherent tomography (OCT) was first applied in biology and medical diagnosis [111,112]. The working principle of OCT is based on Michelson interferometry [113]. The laser light source is divided into two beams through a beam splitter, where one beam illuminates the sample through the focusing lens and part of the light is reflected and received by the detector, while the other beam of light is used as a reference beam, which is reflected by the mirror, and the optical path is adjusted by moving the mirror so that the two beams of light can interfere, and the interference signal is acquired by the detector. The interference signal is post-processed by the computer to generate an interference function, the maximum value of which corresponds to the position of the mirror, and then the subsurface defect profile of the sample is accurately calculated [114,115,116]. Bashkansky et al. [117,118] demonstrated the capability of OCT for assessing SSD in ceramics, achieving detection depths up to 140 μm while also capturing 3D SSD characteristics. Sergeeva et al. [119] employed short-coherence interferometry to evaluate artificially induced SSD in glass, reporting a lateral resolution and depth of 4 and 10 μm, respectively. OCT’s spatial resolution is fundamentally limited by the coherence length of the light source, posing significant challenges for resolving micrometer-scale subsurface crack dimensions. Furthermore, in semiconductor and optical materials, the weak scattering signal originating from SSD often proves insufficient to generate detectable interference patterns [120]. Compounding these issues, noise artifacts arising from surface scattering and reflections can obscure the target signal during data processing [108].

**Figure 10:**
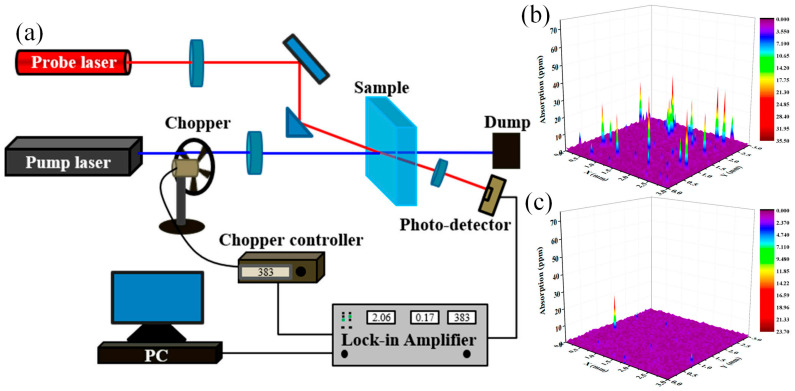
(**a**) Schematic diagram of the PCI experimental setup for photothermal absorption. (**b**,**c**) The 3D absorption mapping of absorbing defects on each sample surface [131].

The absorption property of SSD allows us to non-destructively characterize the SSD of optical components. In recent years, based on the photothermal effect, photothermal absorption has proven to be a new method for the non-destructive detection of SSD and can be used to detect nanoscale contaminants on glass surfaces [121,122,123,124]. The most widely used photothermal absorption methods are photothermal deflection spectroscopy (PDS) and photothermal common-path interferometry (PCI) [125,126]. PCI has been used for the measurement of weak absorption on optical component surfaces [127,128,129], while PDS requires higher laser power and lower sensitivity [130]. A typical schematic diagram of the PCI experimental setup for photothermal absorption measurements is shown in Figure 10a. During PCI measurements, a laser with relatively high power is focused on the measured position of the sample, and then the absorbing defects absorb energy, causing local heating and thermal expansion, leading to a small change in the refractive index. In this case, the interior of the probe laser beam is disturbed, creating interference with the undisturbed outer part. The interference signal is detected by a photodetector and processed by a lock-in amplifier, which further gives the photothermal absorption signal of the sample. Employing PCI-based photothermal spectroscopy, Shi et al. [131] examined the distribution of weak absorption on optical surfaces subjected to various processing techniques. Their findings revealed a strong correlation between the density of defects exhibiting absorption exceeding 2 ppm and subsequent damage performance. Figure 10b presents the corresponding 3D absorption maps of surface defects for each sample. Zhong et al. [132], utilizing photothermal absorption methods to characterize subsurface defects post-IBE treatment, demonstrated that nanoscale defects containing impurities exert a decisive influence on laser damage resistance. Furthermore, analysis of photothermal absorption spectra indicated a reduction in surface weak absorption following wet etching, suggesting that this process can effectively remove absorbing defects [128,133]. To quantitatively model the distribution of absorption defects on fused silica surfaces under diverse processing conditions, Huang et al. [134] introduced a photothermal absorption distribution probability curve based on a normal distribution model. As an emerging detection technique, photothermal absorption offers distinct advantages: it discriminates between bulk and surface absorption within components [135] while providing high sensitivity (<10 ppb), high spatial resolution (<1 μm), non-contact operation, and non-destructive evaluation [136].

#### 3.2.3. Chemical-Based Methods

As a widely used surface characterization technique, X-ray photoelectron spectroscopy (XPS) can provide quantitative information and the chemical status of atoms in the subsurface region. For instance, by using XPS, Sun et al. [137] found that the impurity element defects and SSD on fused silica surfaces can be efficiently removed using the reactive ion etching process. In another study by Shao et al. [138], it was found that the non-bridging oxygen atoms in fused silica could be reduced in the subsurface region after the reactive ion etching process. Köhler et al. [139] found that the amount and chemical binding state of trace contaminants of interest—calcium, cerium, and sodium—originating from various classical optics manufacturing processes, are modified in the subsurface region of fused silica. These studies clearly show that the XPS technique can provide quantitative information on chemical elements and their status in the subsurface region, but depth information is limited, usually less than 10 nm.

Time-of-flight secondary ion mass spectrometry (ToF-SIMS) is a surface-sensitive analytical method that uses a pulsed ion beam to remove molecules from the outermost surface of a sample. Chemical elements are removed from a monolayer of atoms (secondary ions) on the surface, and then accelerated into the “flying tube,” and their mass is determined by measuring the exact time they reach the detector, known as the time of flight [140,141]. ToF-SIMS can provide deeper information about chemical elements in the subsurface region of optical materials, and the penetration depth can be more than hundreds of nanometers [142]. Moreover, the ToF-SIMS technique is reported to provide the 3D distribution of chemical elements [143], which can provide more global information about chemical elements in the subsurface region of optical materials.

IR and Raman spectroscopy are used to obtain SSD information. For instance, Li et al. [123] used IR spectroscopy to detect SSD in fused silica after ion beam etching (IBE), and they found that the Si-O-Si bond angles in the SSD of fused silica decrease, and a densified surface of fused silica can be obtained after ion beam etching. Using Raman spectroscopy, Zhong et al. [132] found that the D1 and D2 defects in fused silica decrease with IBE depth. Micro-Raman spectroscopy has also been used to detect subsurface residual strain, impurities, lattice distortion, and phase transformation [144,145]. More recently, nano-FTIR spectroscopy based on Fourier transform infrared near-field spectroscopy and scattering-type scanning near-field optical microscopy allowed for label-free chemical characterization of optical materials at the nanoscale [146]. For instance, using nanoscale infrared spectroscopy and reactive molecular dynamics simulations, He et al. [147] found that within the elastic deformation region, an elongation of the S-O bond length distribution of fused silica can be revealed upon nanoindentation and nanoscratch tests. Using scattering-type scanning near-field optical microscopy, Yan et al. [148] found that subsurface dislocations, plastic pile-ups, burrs, and microstructures at the subsurface of scratches in silicon caused by single-point diamond scratching can be distinguished. Although these methods are simple and easy to handle, they cannot detect all types of SSD. In addition, these methods generally provide point-by-point detection rather than scanning, which is time-consuming and less efficient.

#### 3.2.4. Other Methods

As a widely used non-destructive technique for interior defect detection, scanning acoustic microscopy (SAM) uses an electrical radio frequency (RF) signal to differentiate surface and subsurface scattering waves and noises, and thus SSD can be detected [149]. However, the lateral resolution of SAM is tens of microns [150], which is not applicable to ultra-precision manufacturing. SSD information is difficult to distinguish since both surface waves and elastic waves are obtained by the detector. Moreover, the SAM method is not applicable to rough surfaces due to the influence of surface scattering waves.

The X-ray diffraction (XRD) method can be used to measure the residual stress in optical materials [151], which is based on the comparison of measured and standard diffraction spectra. The main disadvantage is the limited penetration depth (typically smaller than 20 μm). X-ray computed tomography (CT) can reveal 3D information on SSD in optical materials [152], but the main drawback of CT methods is X-ray radiation, which is hazardous to the operator.

Currently, the main technical challenges researchers face in detecting or quantifying SSD involve balancing resolution with non-destructiveness, overcoming material-specific obscuration layers, and establishing traceable quantification standards. While destructive methods remain the gold standard for critical validations, advances in computational optics and multi-sensor fusion present promising pathways toward practical non-destructive solutions. Note that both destructive and non-destructive methods are relatively reliable and provide useful SSD information for optical materials. However, there are some disadvantages that need to be overcome in future studies. A summary and comparison of these methods can be found in Table 1.

## 4. Summary

In this paper, methods of characterizing SSD in optical materials are reviewed. SSD in optical materials can be exposed after physical or chemical treatment, and destructive methods can provide direct, reliable, and quantitative characterization of the SSD topography; however, they cannot provide global information on SSD, and they are time-consuming processes. In contrast, using the physical and chemical properties of SSD, non-destructive methods are low-cost and efficient, and they characterize the SSD indirectly. Current SSD characterization methods are aimed at small-sized optical materials; applying them to larger-sized optical materials or developing new SSD characterization methods with higher measurement accuracy and more global information will be the topics of future studies.

## Figures and Tables

**Table 1:** Comparison of various SSD detection methods presented in this paper.

Methods	Resolution	Measurand	Advantages	Disadvantages
Mechanical-based methods	nm and μm, depending on testing conditions	Elastic modulus, hardness, …	Simple and easy to operate	Measurement errors are relatively high; cannot provide global distribution of SSD
Polishing-based methods	nm/μm, depending on observation methods	SSD topography	Simple and easy to operate	Measurement errors are relatively high; cannot provide global distribution of SSD
Bonded-interface methods	nm/μm, depending on observation methods	SSD topography	Direct observation of SSD	Not applicable to hard and brittle materials
Ball-machining methods	nm/μm, depending on observation methods	SSD topography	Direct observation of SSD	Measurement errors are relatively high
Chemical etching	nm/μm depths, depending on observation methods	SSD topography	Easy to operate with low cost	Measurement errors are relatively high; chemicals detrimental to humans and environments
Numerical predictive methods	nm/μm depths	SSD depth	Simple, rapid, and low cost	Unable to predict 3D configuration and SSD distribution; poor universality
TRIM	<20 μm longitudinal resolution	Scattering light from SSD	Global scanning and in-process detection	Hard to measure SSD depth quantitatively; high requirement for surface condition, suitable for polished samples
CLSM	Lateral and longitudinal resolution of 3D images can reach 0.1 μm and 50 nm, respectively	Light emitted from SSD	Global scanning and applicable to 3D configuration of SSD	High requirement for surface condition, suitable for polished samples; can be influenced by surface scattering
OCT	Lateral and longitudinal resolution can reach 4 μm and 10 nm, respectively	Light interference signal from SSD	Global scanning and applicable to 3D configuration of SSD	Cannot detect low light-scattering materials; difficult to process images; can be influenced by surface scattering
DFCM	Longitudinal resolution of 3D images can reach 60 nm	Scattering light from SSD	Global scanning and applicable to 3D configuration of SSD	High requirement for surface condition, suitable for polished samples, such as large or curved optical components
PCI	Spatial resolution < 1 μm	Refractive index	High sensitivity (<10 ppb), non-contact	High requirement for surface condition, suitable for polished samples
XPS	Spatial resolution can reach 1 nm	Chemical composition	High sensitivity	Sample size is small; high requirement for surface condition, suitable for polished samples
ToF-SIMS	Spatial resolution can reach 1 nm	Chemical composition	High sensitivity, global scanning, and applicable to 3D configuration of SSD composition	Sample size is small; high requirement for surface condition, suitable for polished samples
IR	~1 μm, can reach ~10 nm for nano-FTIR	Chemical structure	Chemical identification, non-contact	High requirement for surface condition, suitable for polished samples
Raman	~1 μm	Chemical structure	Chemical identification, non-contact	High requirement for surface condition, suitable for polished samples

## Data Availability

No new data were created or analyzed in this study. Data sharing is not applicable to this article.
